# Anxiolytic-Like Effects of Antisauvagine-30 in Mice Are Not Mediated by CRF_2_ Receptors

**DOI:** 10.1371/journal.pone.0063942

**Published:** 2013-08-28

**Authors:** Eric P. Zorrilla, Amanda J. Roberts, Jean E. Rivier, George F. Koob

**Affiliations:** 1 Committee on the Neurobiology of Addictive Disorders, The Scripps Research Institute, La Jolla, California, United States of America; 2 Molecular and Integrative Neurosciences Department, The Scripps Research Institute, La Jolla, California, United States of America; 3 The Clayton Foundation Laboratories for Peptide Biology and Structural Biology Laboratory, The Salk Institute for Biological Studies, La Jolla, California, United States of America; 4 Department of Neurosciences, University of California San Diego, La Jolla, California, United States of America; Medical University of South Carolina, United States of America

## Abstract

The role of brain corticotropin-releasing factor type 2 (CRF_2_) receptors in behavioral stress responses remains controversial. Conflicting findings suggest pro-stress, anti-stress or no effects of impeding CRF_2_ signaling. Previous studies have used antisauvagine-30 as a selective CRF_2_ antagonist. The present study tested the hypotheses that 1) potential anxiolytic-like actions of intracerebroventricular (i.c.v.) administration of antisauvagine-30 also are present in mice lacking CRF_2_ receptors and 2) potential anxiolytic-like effects of antisauvagine-30 are not shared by the more selective CRF_2_ antagonist astressin_2_-B. Cannulated, male CRF_2_ receptor knockout (*n* = 22) and wildtype littermate mice (*n* = 21) backcrossed onto a C57BL/6J genetic background were tested in the marble burying, elevated plus-maze, and shock-induced freezing tests following pretreatment (i.c.v.) with vehicle, antisauvagine-30 or astressin_2_-B. Antisauvagine-30 reduced shock-induced freezing equally in wildtype and CRF_2_ knockout mice. In contrast, neither astressin_2_-B nor CRF_2_ genotype influenced shock-induced freezing. Neither CRF antagonist nor CRF_2_ genotype influenced anxiety-like behavior in the plus-maze or marble burying tests. A literature review showed that the typical antisauvagine-30 concentration infused in previous intracranial studies (∼1 mM) was 3 orders greater than its *IC_50_* to block CRF_1_-mediated cAMP responses and 4 orders greater than its binding constants (*K_d_*, *K_i_*) for CRF_1_ receptors. Thus, increasing, previously used doses of antisauvagine-30 also exert non-CRF_2_-mediated effects, perhaps via CRF_1_. The results do not support the hypothesis that brain CRF_2_ receptors tonically promote anxiogenic-like behavior. Utilization of CRF_2_ antagonists, such as astressin_2_-B, at doses that are more subtype-selective, can better clarify the significance of brain CRF_2_ systems in stress-related behavior.

## Introduction

In mammals, the stress-related peptide corticotropin-releasing factor (CRF) and its paralogs urocortins 1, 2, and 3 (Ucn 1, Ucn 2, Ucn 3), activate two CRF receptor subtypes, CRF_1_ and CRF_2_, to varying degrees [1]. CRF_1_ receptors mediate endocrine, behavioral, and autonomic responses to stress, which has spurred the development of drug-like CRF_1_ antagonists [2]. In contrast, the role of brain CRF_2_ receptors in stress responses remains controversial. Studies have implicated anti-stress-like actions, pro-stress-like actions, or a lack of involvement of CRF_2_ receptors [1]. Part of this uncertainty may reflect that, unlike the case with CRF_1_ antagonists [2], highly selective (>10,000-fold selectivity), small molecule CRF_2_ antagonists remain unavailable. Researchers have instead used truncated CRF_2_-preferring (100–1000-fold selectivity) peptide fragments as CRF_2_ antagonists, principally [D-Phe^11^,His^12^]sauvagine(11–40)NH_2_ (antisauvagine-30; [3] and cyclo(31–34)[D-Phe^11^,His^12^,CαMeLeu^13,39^,Nle^17^,Glu^31^,Lys^34^]Ac-sauvagine_(8–40)_ (astressin_2_-B; [4]).

Antisauvagine-30 has been described as a selective CRF_2_ antagonist in the literature (1530 hits in Google Scholar as of August 2012). Antisauvagine-30 potently displaces radioiodinated CRF-related ligands from HEK293 cell membranes expressing recombinant mCRF_2b_ (*K_d_* = 1.4 nM; [3], hCRF_2a_ (*K_i_* = 0.8 nM; [5], or mCRF_2b_ receptors (*K_i_* = 0.41 nM; [6] and has lower affinity for HEK293 membranes expressing CRF_1_ receptors. Several findings suggest, however, that antisauvagine-30 may block CRF_1_ receptors at doses that have been used *in vivo*. First, antisauvagine-30 can displace [^125^I]-oCRF from HEK293-rCRF_1_ membranes (*Ki* = 154–166 nM; [3], [6] and [^125^I]-sauvagine from HEK293-hCRF_1_ membranes (*Ki* = 100 nM; [7]). Similarly, antisauvagine-30 competes with [^125^I]-astressin to bind rat and human uncoupled CRF_1_ receptors (*Ki* = 66 and 170 nM; [7], [8]. Yet, many intracerebroventricular and intracerebral studies have infused antisauvagine-30 at ∼4 orders greater concentrations (1–2 mM) (e.g., see Table 1). Moreover, in its original characterization, antisauvagine-30 showed ∼30% of the rCRF_1_ antagonist potency of astressin [3], a potent CRF_1_ antagonist. Accordingly, antisauvagine-30 blocks oCRF-induced cAMP accumulation in HEK293-rCRF1 cells [9] and oCRF-induced cAMP responses in human retinoblastoma Y79 cells [10] with *IC_50s_* = 1–2 µM, concentrations 3 orders lower than those that have been injected. The incomplete selectivity of antisauvagine-30 raises concern that some putative anxiolytic/anti-stress-like actions of antisauvagine-30 previously attributed to antagonism of brain CRF_2_ receptors may involve a non-CRF_2_ target, such as CRF_1_ receptors.

**Table 1:** Intracerebroventricular (ICV) studies of antisauvagine-30 effects on stress- or anxiety-related endpoints.

Reference	Minimum effective ICV injection	Concentration (µM)	Dose (nmol)	Result
[14]	400 ng/0.5 µl	219	0.11	INCREASED anxiety-like behavior
[36]	1–5 µg/2 µl	140–680	∼0.27–1.37	Reduced anxiety-like behavior
[37]	1–10 nmol/2.5 µl	400–4000	1–10	Reduced CRF-induced anxiety-like behavior and anorexia
[38]	2.2 nmol/2 µl	1100	2.2	Reduced stress-induced weight loss
[39]	10 µg/5 µl	550	2.7	Reduced stress-induced deficits in prepulse inhibition of startle
[40]	10 µg/2 µl	1370	2.7	Reduced burn-induced hypermetabolism
[41]	10 µg/2 µl	1370	2.7	Reduced Ucn 2-induced neuroactivation
[42]	3 nmol/5 µl	600	3	Reduced CRF-induced startle and prepulse inhibition deficits
[43]	3 nmol/5 µl	600	3	Reduced CRF-induced startle
[44]	3 nmol/5 µl	600	3	Reduced anxiety-like behavior
[45]	20 µg/5 µl	1100	5.5	Reduced CRF- and stress-induced neuroactivation
[46]	20 µg/5 µl	1100	5.5	Reduced stress-induced anorexia
[47]	20 µg/5 µl	1100	5.5	Reduced stress- and Ucn 2/Ucn 3-induced HPA-activation
[48]	20 µg/3 µl	1800	5.5	Reduced somatic and noradrenergic responses to opiate withdrawal
[49]	20 µg/3 µl	1800	5.5	Reduced acquisition of conditioned defeat
[50]	6 nmol/10 µl	600	6	Reduced *des*-acyl ghrelin-induced changes in gastric motor activity
[51]	Osmotic minipump	1200	30/day	Reduced CRF/Ucn 1-induced anorexia and weight loss

Note: Not only doses, but also concentrations, are listed because relative dilution of the injected concentration across a given volume of brain is what will determine the local concentration relevant to receptor pharmacodynamics. CRF = corticotropin-releasing factor, HPA = hypothalamic-pituitary-adrenal axis, Ucn = urocortin.

Many antibodies [11] and antagonists [12] were subsequently found to have off-target binding or activity when evaluated in knockout (KO) mice. Here, we tested the hypotheses that any potential anxiolytic-like actions of antisauvagine-30 would 1) be present in mice lacking functional CRF_2_ receptors, and 2) not be shared by the more selective CRF_2_ antagonist astressin_2_-B. Astressin_2_-B binds to CRF_2_ receptors *in vitro* with similar potency as does anti-sauvagine-30 (e.g., displacement of [^125^I]sauvagine from CHO-hCRF_2a_ membranes (*Ki* = 0.49 vs 0.29 nM), from intrinsic rCRF_2b_ in A7r5 cells (*Ki* = 0.17 vs 0.77 nM), and from CRF_2a_ in rat olfactory bulb (*Ki* = 0.50 vs 0.84 nM) [8]. But, astressin_2_-B shows one order less affinity for CRF_1_ receptors (*Ki*>1000 nM and 890 nM, respectively) [7], [8] than does antisavuagine-30 (*Ki* = 100 nM) [3], [6], [7], [8].

A secondary goal of the present study was to evaluate the anxiety-related phenotype of CRF_2_ KO mice backcrossed to C57BL/6J background. Previous studies that reported an anxiogenic-like phenotype of CRF_2_ knockout mice were performed on a hybrid 129SvJ-C57BL/6J genetic background [13], [14]. However, mixed genetic background transgenic mice can lead to spurious or inconsistent results due to the confounding (due to genetic linkage) and interactive influence of mixed genetic background on observed phenotypes [15]. The CRF_2_ null mutation was introduced into embryonic stem cells of the 129Sv genetic background. Due to genetic linkage, CRF_2_ null mutant mice studied on a hybrid background will overrepresent the 129Sv genetic background as compared to wildtype mice, which will show comparatively more C57BL/6 background [15]. Anxiogenic-like behavior is greater in 129Sv strain mice than in C57BL/6 mice, however [16], [17], [18], [19]. As a result, it is not clear whether the previously reported anxiogenic-like CRF_2_ KO phenotype is actually due to the null mutation as opposed to linked 129Sv genetic material. Potentially consistent with the latter possibility, no anxiogenic-like phenotype in elevated plus-maze or open field behavior was seen in CRF_2_ KO mice backcrossed 3 generations (∼87.5%) to a C57BL/6J background (Coste et al., 2000). Therefore, we here revisit the anxiety-related phenotype of CRF_2_ KO mice that were previously reported to show anxiogenic-like behavior on a hybrid background [13], but now studied after being backcrossed extensively (>99.975%) onto a C57BL/6J background.

## Materials and Methods

### Ethics Statement

Procedures adhered to the National Institutes of Health Guide for the Care and Use of Laboratory Animals (NIH publication no. 85–23, 1996) and Principles of Laboratory Animal Care and were approved by the Institutional Animal Care and Use Committee of The Scripps Research Institute (protocol #08-0010). All surgery was performed under isoflurane anesthesia, and all efforts were made to minimize suffering.

### Subjects

Subjects were adult (26.5–32.3 g at study onset), male CRF_2_ receptor KO (*n* = 22; *Crhr2^tm1Klee^*/*Crhr2^tm1Klee^*; [13] and wildtype littermate mice (*n* = 21; WT, ≥12 generations C57BL/6J backcrossing; ≥99.9755869% consomy) offspring of heterozygote breeding. Mice were group-housed under a reverse 12 h/12 h light/dark cycle in a humidity- (60%) and temperature-controlled (22°C) vivarium with chow (LM-485 Diet 7012, Harlan, Madison, WI) and water available *ad libitum*.

### Surgery

Anaesthetized (isoflurane, 1–3%) mice were stereotaxically (David Kopf, Tujunga, CA) implanted with a 27-gauge, 7.5 mm stainless steel guide cannula 1 mm above the lateral ventricle. Coordinates (in mm) were (anterior/posterior: −0.1, medial/lateral: ±1.0 from bregma, dorsal/ventral: −1.5 from skull; [20]. A 30-gauge obturator maintained patency. Mice recovered ≥7 days before testing. Cannula placement was inferred from successful gravity injection and from ventricular spread of injected dye in randomly tested mice.

### Drugs and injection

Antisauvagine-30 and astressin_2_-B were synthesized using solid-phase methodology, purified using HPLC and characterized using capillary zone electrophoresis, HPLC and MS [4]. Peptides were dissolved in 0.5× PBS before testing and kept on ice. For intracerebroventricular (i.c.v.) infusions, the 30-gauge injector extended 1 mm beyond the cannula and was attached to tubing (0.01 i.d., 0.03 o.d. inches) from which 2 µl solution was delivered into the ventricle by gravity over 30 sec. The injector was left in place for 60 sec. The pretreatment intervals, during which the mouse was returned to its home cage were 15 min for the marble burying test and 30 min for the plus-maze and shock-induced freezing tests.

### Study design

Mice were tested during the dark phase in the marble burying, elevated plus-maze, and shock-induced freezing tests using a between-subjects design for treatment. The same set of mice were subjects in the 3 tests. Experiments involved a 2 (Genotype: WT vs. KO)×3 (Antagonist: vehicle vs. antisauvagine-30 vs. astressin_2_-B) factorial design. The dose of antisauvagine-30 (i.c.v. ∼3 nmol, or 10.7 µg) was representative of doses used in previous studies of stress- or anxiety-related endpoints (Table 1). Astressin_2_-B was administered at the same dose. Tests were spaced by one week, and mice received a given drug treatment no more than twice across the three tests.

### Marble burying

For marble burying testing [21], mice were individually placed in a polycarbonate cage (29×18×12 cm) containing 20 marbles (1.5 cm diameter) evenly spaced on 5-cm deep bedding. Marbles covered at least two-thirds by bedding, an index of anxiogenic-like behavior, were counted 30 min later.

### Elevated plus-maze

The plus-maze apparatus has four arms (5×30 cm) at right angles to each other, elevated 30 cm from the floor. Two arms have 16-cm black plastic walls (closed arms), and two arms have 16-cm clear plastic walls (more open arms). Controls tested in this modified apparatus spend 35–40% of their time on the open arms, allowing changes to be detected bidirectionally; mice tested in the original plus-maze (open arms with no wall) typically spend 10–15% of their time on the open arms, making it difficult to detect anxiogenic-like effects. Mice were placed on the center of the maze, and behavior was videorecorded for 5 min. Decreases in % open arm time, calculated as: 100*open arm time/(open arm time+closed arm time) [22], indicate increased anxiety-like behavior. More total arm entries indicate increased locomotor activity [22].

### Shock-induced freezing

Mice were placed in a Mouse NIR Video Fear Conditioning System (Med Associates, St. Albans, VT) housed in a soundproofed box, allowed to habituate for 2 min and then exposed to three 1.5 mA, 1-sec footshocks, separated by 20 sec. Freezing, a CRF/CRF_1_-dependent defensive response [23], was measured automatically from real-time video recordings (30 frames per second) across 15 min using Video Fear Conditioning Software (Med Associates) that distinguishes between subtle movements, such as whisker twitches, tail flicks and freezing behavior.

### Statistics

Analysis of variance (ANOVA) was used to evaluate effects of Genotype, Antagonist and their interaction. Fisher's protected least significant difference tests identified pairwise differences. The software used was Systat 12.0 (SPSS, Chicago, IL).

## Results

**Figure 1:**
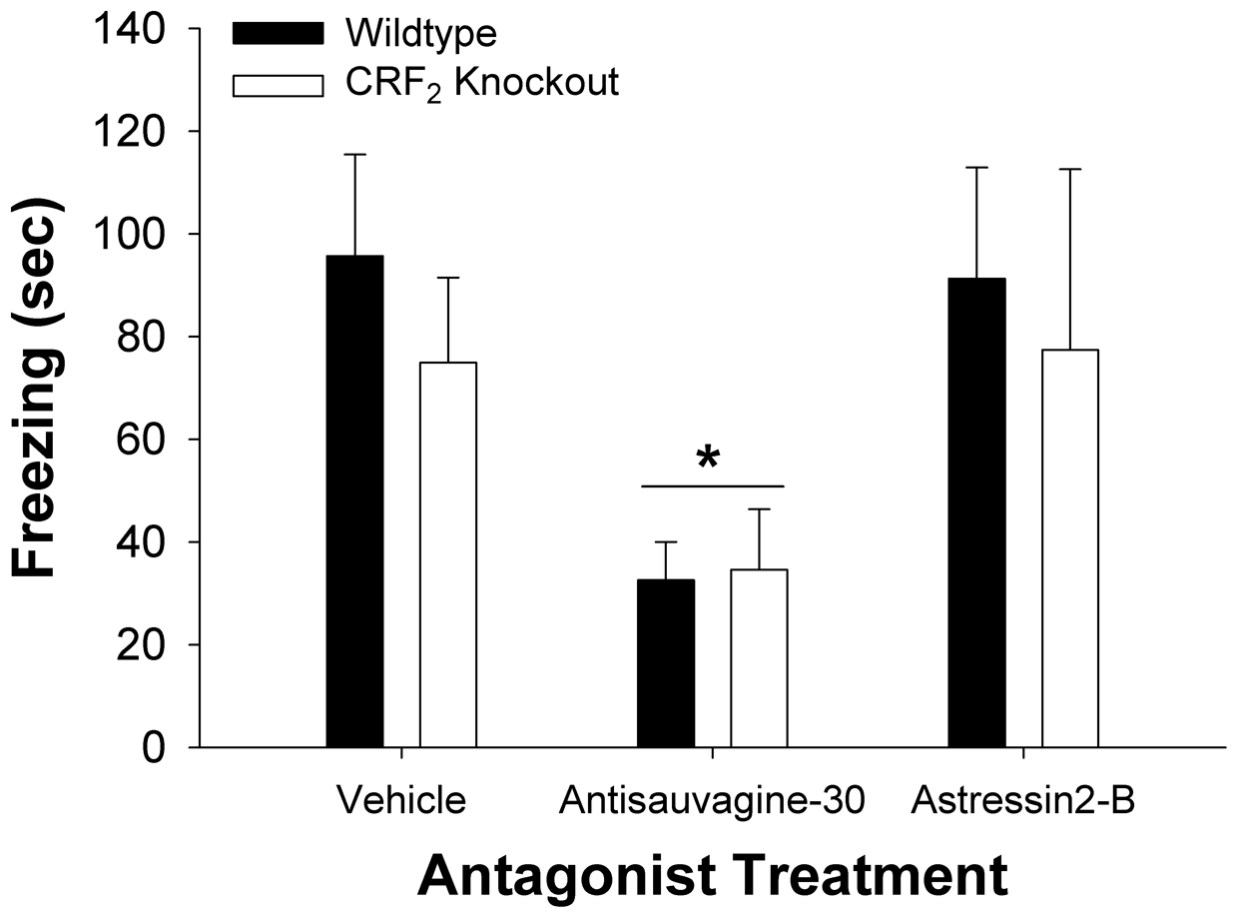
Effects of antisauvagine-30, astressin_2_-B and CRF_2_ genotype on shock-induced freezing. The data are expressed as *M* ± SEM. Antisauvagine-30 (i.c.v., 3 nmol) significantly and equally reduced the duration of shock-induced freezing in both wildtype and CRF_2_ knockout mice. In contrast, the same dose of astressin_2_B, a selective CRF_2_ antagonist, and CRF_2_ null genotype did not alter shock-induced freezing (*n* = 6–9/group). *p<0.05, differs from vehicle and astressin_2_-B-treated mice (Fisher's protected least significant difference test).

Figure 1 shows that antisauvagine-30 reduced the duration of shock-induced freezing in both WT and CRF_2_ KO mice (Antagonist: *F*_2,37_ = 4.17, *p*<0.05). Antisauvagine-30-treated mice froze less than mice pretreated with either vehicle or astressin_2_-B (*p*s<0.05), which did not differ from one another (*p* = 0.96). No Genotype (*F*_1,37_ = 0.03, *p*>0.85) or Genotype×Antagonist effects (*F*_2,37_ = 0.39, *p*>0.68) were seen.

Table 2 shows that there were no significant Genotype, Antagonist or Genotype×Antagonist effects on raw open arm time (*F*_1,37_ = 1.07, *F*_2,37_ = 1.22, *F*_2,37_ = 2.41, all *p*s>0.1), % open arm time calculated as a function of total arm time (*F*_1,37_ = 1.12, *F*_2,37_ = 1.16, *F*_2,37_ = 2.13, all *p*s>0.1), or the total number of arm entries in the elevated plus-maze (*F*_1,37_ = 0.42, *F*_2,37_ = 1.54, *F*_2,37_ = 0.15, all *p*s>0.2). There also were no significant Genotype (*F*_1,37_ = 0.35, *p*>0.55), Antagonist (*F*_2,37_ = 0.12, *p*s>0.89) or Genotype×Antagonist (*F*_2,37_ = 1.52, *p*>0.23) effects on the number of marbles buried in the marble burying test. *A priori* analysis in vehicle-treated mice considered separately also indicated no significant Genotype effect on shock-induced freezing (*p*>0.15); plus maze measures of % open arm time (*p*>0.15), open arm time (*p*>0.14), or total arm entries (*p*>0.72); or marbles buried (*p*>0.24).

**Table 2:** Effects of genotype and CRF antagonist on behavior in the elevated plus-maze and marble burying tests.

	Vehicle		Antisauvagine-30		Astressin_2_-B	
	Wildtype (*n* = 9)	CRF_2_ KO (*n* = 8)	Wildtype (*n* = 6)	CRF_2_ KO (*n* = 7)	Wild type (*n* = 6)	CRF_2_ KO (*n* = 7)
*Elevated plus-maze*						
Open arm time, %	41.1±12.7	18.2±8.0	27.4±9.0	9.4±2.3	25.9±8.0	41.0±11.7
Open arm time, sec	116±37	48±21	71±24	22±5	64±24	110±32
Total arm entries	10.8±2.1	11.9±2.1	15.7±2.6	15.7±2.1	11.8±2.1	14.7±3.7
*Marble burying*						
Marbles buried	9.9±2.7	11.2±1.8	11.5±2.8	10.0±2.4	6.5±2.8	10.7±2.1

The data are expressed as *M*+SEM. KO = knockout.

Table 3 lists published studies in which antisauvagine-30 was administered site-specifically to discrete brain regions as a CRF_2_ antagonist. As can be seen, the concentrations that have been infused locally range from 137–2000 µM, on the order of those given i.c.v. previously (Table 1) and in the present study. The median concentration infused, 1050 µM is ∼3 orders greater than the reviewed *IC_50_* of antisavuagine-30 to block CRF_1_-mediated cAMP responses (∼1–2 µM) and ∼4 orders greater than reviewed binding constants (*K_d_*, *K_i_*∼0.066–0.166 µM) of antisauvagine-30 for CRF_1_ receptors.

**Table 3:** Intracerebral (IC) site-specific studies of antisauvagine-30 effects on stress- or anxiety-related endpoints.

Reference	Minimum effective intracerebral injection	Dose (pmol)	Concentration (µM)	Result
[52]	0.25 µg/0.5 µl	68.5	137	Reduced alcohol-induced increases in dynorphin levels
[53]	0.25 µg/0.5 µl	68.5	137	Reduced alcohol-induced increases in β-endorphin levels
[54]	0.4 µg/0.5 µl	110	220	Reduced stress-enhanced fear conditioning and Mek-1/2-dependent signaling
[55]	0.4 µg/0.5 µl	110	220	Reduced stress/CRF-induced anxiety-like behavior and cognitive deficits
[56]	0.4 µg/0.5 µl	110	220	Reduced stress-induced anxiety-like behavior and fear conditioning deficits
[57]	0.2 nmol/0.5 µl	200	400	Reduced acquisition of a CRF-induced conditioned place aversion
[58]	0.5 µg/0.2 µl	137	685	Reduced the expression of conditioned defeat
[59]	0.5 nmol/0.5 µl	500	1000	Reduced inescapable shock-induced shuttlebox escape deficits
[60]	55 pmol/0.05 µl	55	1100	Reduced ethanol-induced hypothermia
[61]	2 µg/0.5 µl	550	1100	Reduced isolation-induced anxiety-like behavior
[62]	2 µg/0.5 µl	550	1100	Reduced CRF-induced CeA serotonin efflux in amphetamine pre-treated rats
[63]	2 µg/0.5 µl	550	1100	Reduced heightened anxiety-like behavior in amphetamine pre-treated rats
[64]	2 µg/0.5 µl	550	1100	Reduced CRF- or CeA-activation-induced mPFC serotonin efflux
[65]	2 µg/0.5 µl	550	1100	Reduced CRF-induced increases in NAc serotonin efflux
[66]	1 nmol/0.5 µl	1000	2000	Reduced stress-induced anorexia
[67]	1 nmol/0.5 µl	1000	2000	Reduced Ucn 2-induced BLA serotonin efflux and neuroactivation.

Note: Not only doses, but also concentrations, are listed because relative dilution of the injected concentration across a given volume of brain is what will determine the local concentration relevant to receptor pharmacodynamics. Mek-1/2 = Mitogen-activated extracellular signal-regulated kinases; CeA = central nucleus of the amygdala; BLA = basolateral amygdala; mPFC = medial prefrontal cortex; NAc = nucleus accumbens; CRF = corticotropin-releasing factor; Ucn 2 = urocortin 2.

## Discussion

The present study found that i.c.v. infusion of a dose of antisauvagine-30 intermediate to those used in the literature reduced shock-induced freezing in both wild-type and CRF_2_ KO mice, unlike the CRF_2_ antagonist astressin_2_-B, which did not mitigate shock-induced freezing in either genotype. The present study also found that neither CRF_2_ KO nor i.c.v. astressin_2_-B infusion produced anxiolytic-like effects in 3 tests of anxiety-like behavior. Altogether, the results indicate that increasing doses of antisauvagine-30 lose their specificity and can exert non-CRF_2_-mediated effects at doses previously used. The collective results do not support the hypothesis that activation of brain CRF_2_ receptors tonically promotes anxiogenic-like behavior.

Antagonism of CRF_1_ receptors is a plausible mechanism for the non-CRF_2_ mediated anxiolytic-like actions of antisauvagine-30 seen here on shock-induced freezing. The low-moderate CRF_1_ binding affinities (∼100 nM) of antisauvagine-30 are not shared by the other widely used CRF_2_ antagonist, astressin_2_-B (*K_i_*>1000 nM and 890 nM, respectively; [7], [8], which is similarly potent to antisauvagine-30 at binding CRF_2_ receptors (e.g., displacement of [^125^I]sauvagine from CHO-hCRF_2a_ membranes [*K_i_* = 0.49 vs. 0.29 nM], from intrinsic rCRF_2b_ in A7r5 cells [*K_i_* = 0.17 vs. 0.77 nM], and from CRF_2a_ in rat olfactory bulb [*K_i_* = 0.50 vs. 0.84 nM]; [8]. Accordingly, the i.c.v. dose of astressin_2_-B used here, which can block anorexia induced by urocortin 3, a selective CRF_2_ agonist [24], did not reduce shock-induced freezing. The results suggest that astressin_2_-B is more CRF_2_-selective than antisauvagine-30.

Many previous studies using antisauvagine-30 have interpreted that its effects were not CRF_1_ mediated because central administration of small molecule, selective CRF_1_ antagonists did not produce the same effects. Unfortunately, these comparisons have involved excessively lipophilic CRF_1_ antagonists, such as NBI27914, CP-154,526, or antalarmin, which are water insoluble, precipitate upon central administration and may therefore not diffuse to target sites or be available for pharmacological activity. Better controls would involve less hydrophobic, recently developed CRF_1_ antagonists more suitable for intracerebral administration, such as NBI-35965, GW-876008, pexacerfont or BMS-561,388.

Neither CRF_2_ KO nor selective CRF_2_ antagonism via astressin_2_-B altered behavior in three anxiety models, suggesting that CRF_2_ signaling is not a key modulator of anxiety-like behavior under basal conditions. Two previous studies that reported a basal anxiogenic-like phenotype of CRF_2_ knockout mice were performed on a hybrid 129SvJ-C57BL/6J genetic background [13], [14]. In contrast, similar to the present results in mice fully backcrossed onto a C57BL/6J background, no significant anxiety-like phenotype was seen in CRF_2_ knockout mice backcrossed 3 generations toward a C57BL/6J background [25]. Thus, because 129Sv and C57BL/6J mice differ in anxiety-like behavior [16], [17], [18], [19], genetic background may have interacted with the effect of CRF_2_ null mutation on behavioral measures in previous studies [15]. However, these results should not be prematurely concluded to mean that CRF_2_ receptors do not modulate anxiety-like behavior. Consistent with an anxiolytic-like action of CRF_2_ activation, i.c.v. administration of type 2 urocortins, selective CRF_2_ agonists, can produce anxiolytic-like and anti-stress-like behavioral effects [26], [27], [28], [29], [30], [31], [32], [33], [34]. Perhaps CRF_2_ receptors are normally quiescent under basal conditions, but are recruited in compensatory opposition to high or more sustained stress, as brought out following stressors or the anxiogenic-like 129Sv genetic background. Consistent with this hypothesis, CRF_2_ KO mice previously showed an anxiogenic-like phenotype in the light-dark box test following 30-min immobilization stress, but not under basal conditions (see Fig. 6A in [35]). Under this view, the stressful aspects of the 3 tests used in the present study (novelty, brief shock) may have been too brief in duration (<5 min), mild in magnitude, or initiated too soon before the behavioral assessment to allow a putative compensatory CRF_2_ response to be observed. Finally, it cannot be ruled out that a larger sample size might have led to a statistically significant *p*-value. For example, a trend for an anxiogenic-like effect of CRF_2_ null mutation, as reported previously [13], [14], was present in vehicle-treated subjects of the elevated plus-maze that, if considered separately, would have attained significance with a sample size of 16/group (standardized Cohen's *d* = −0.73).

While antisauvagine-30 exerted non-CRF_2_ actions at the tested dose, this does not mean that it is intrinsically non-selective. Lower *in vivo* doses or concentrations might be shown via a KO control study to be adequately selective for functional studies. Indeed, the finding that a low central dose of antisauvagine-30 (i.c.v., 400 ng) previously produced an anxiogenic-like effect, opposite to those seen with increasing doses of the antagonist (see Table 1), is consistent with the interpretation that antisauvagine-30 may lose specificity with increasing doses. The present result with a 3 nmol dose of antisauvagine-30 suggests that many (if not most) previous intracranial administration studies used a dose that can exert non-CRF_2_ mediated effects, complicating their interpretation (Table 1). Utilization of CRF_2_ antagonists at doses validated to be subtype-selective in knockout mice can help further clarify the biological significance of brain CRF_2_ systems in stress-related behavior.
